# A Network Pharmacology Approach to Uncover the Multiple Mechanisms of* Hedyotis diffusa* Willd. on Colorectal Cancer

**DOI:** 10.1155/2018/6517034

**Published:** 2018-02-12

**Authors:** Xinkui Liu, Jiarui Wu, Dan Zhang, Kaihuan Wang, Xiaojiao Duan, Xiaomeng Zhang

**Affiliations:** Department of Clinical Chinese Pharmacy, School of Chinese Materia Medica, Beijing University of Chinese Medicine, Beijing 100102, China

## Abstract

**Background:**

As one of the most frequently diagnosed cancer diseases globally, colorectal cancer (CRC) remains an important cause of cancer-related death. Although the traditional Chinese herb* Hedyotis diffusa* Willd. (HDW) has been proven to be effective for treating CRC in clinical practice, its definite mechanisms have not been completely deciphered.

**Objective:**

The aim of our research is to systematically explore the multiple mechanisms of HDW on CRC.

**Methods:**

This study adopted the network pharmacology approach, which was mainly composed of active component gathering, target prediction, CRC gene collection, network analysis, and gene enrichment analysis.

**Results:**

The network analysis showed that 10 targets might be the therapeutic targets of HDW on CRC, namely, HRAS, PIK3CA, KRAS, TP53, APC, BRAF, GSK3B, CDK2, AKT1, and RAF1. The gene enrichment analysis implied that HDW probably benefits patients with CRC by modulating pathways related to cancers, infectious diseases, endocrine system, immune system, nervous system, signal transduction, cellular community, and cell motility.

**Conclusions:**

This study partially verified and predicted the pharmacological and molecular mechanism of HDW against CRC from a holistic perspective, which will also lay a foundation for the further experimental research and clinical rational application of HDW.

## 1. Introduction

Colorectal cancer (CRC) is a multifactorial disease concerning environmental, lifestyle, genetic, or other risk factors [1], and it has posed a formidable potential threat to public health owing to its high morbidity and mortality [2]. Treatment strategies for CRC include surgery, chemotherapy, radiotherapy, targeted therapies, and immunotherapy [3–5]. The therapies for CRC have been well developed in recent decades [6]; nevertheless, its mortality remains relatively high as a result of frequent recurrence and metastasis [4]. The main therapeutic option for CRC is chemotherapy, and appropriate chemotherapy approaches effectively prolong the life expectancy and improve the performance status of patients with CRC [7, 8]. However, the application of chemotherapy for CRC is largely limited by its fearful side effects and drug resistance [4]. Take FOLFOX (oxaliplatin, 5-fluorouracil, and leucovorin) as an example. FOLFOX is one of the most prevalent chemotherapy regimens and is also a standard first-line treatment strategy for CRC [9, 10]. Even so, when patients with CRC are treated with FOLFOX, a variety of side effects often occur, such as bone marrow suppression, gastrointestinal reaction, and abnormal liver function [11]. Given this, more effective and less toxic therapies are desperately needed for treating CRC [12].

As a well renowned traditional Chinese folk medicine,* Hedyotis diffusa* Willd. (HDW) belongs to the Rubiaceae family and is a natural herbal remedy usually found in the orient and tropical Asia in countries such as China, Japan, and Indonesia [13, 14]. In terms of traditional Chinese medicine (TCM) theory, HDW possesses heat-clearing, detoxification, promotion of blood circulation, and removal of blood stasis effects [15]. HDW has long been extensively utilized in several Chinese medicine formulae to clinically treat inflammatory and infectious diseases like sore throat, bronchitis, hepatitis, urethritis, and appendicitis [16–20]. Moreover, HDW has also been used as an adjuvant therapy for the treatment of certain malignancies, including colorectal, liver, stomach, lung, and breast cancers, with relatively fewer and milder side effects [18, 21–30]. It has been reported that HDW displays an array of pharmacological effects, including antioxidant, anti-inflammatory, antibacterial, antiangiogenic, proapoptotic, and immunomodulatory activities [14, 31–35]. And, more importantly, a growing number of preclinical cancer studies demonstrate that HDW exhibits striking anticancer activity [32, 36–38]. However, the potential mechanisms of its antitumor effect have not been completely elucidated.

TCM is a multicomponent, multitarget, and multipathway treatment that realizes its particular therapeutic efficacy by modulating the biological network of body systems [39], and thereby it is relatively difficult to detect the accurate mechanisms of TCM solely by conventional experimental method [40]. Consequently, the new and appropriate approaches are urgently needed to systematically and comprehensively dissect the mechanisms of herbal medicines [41]. Owing to the rapid advancement of bioinformatics, the network pharmacology has become an emerging approach to efficiently and systemically disclose the molecular and pharmacological mechanisms of TCM [42, 43]. Unlike earlier reductionist “one drug, one target” means, network pharmacology focuses on the fact that numerous active ingredients interact with multiple diverse genes or proteins, highlighting a holistic thought also shared by TCM [44, 45]. Network pharmacology can reflect and clarify the interactive relationship between multiple drugs, multiple targets, and multiple diseases. Meanwhile, it abstracts the relationship into a network model and illustrates the action of drugs on human biological network from a systematic perspective [43]. Thus, we select the network pharmacology approach to explore the impact of HDW on CRC to clarify its medical value.

## 2. Materials and Methods

### 2.1. Chemical Compounds in HDW

To collect the compounds of HDW, we used the Traditional Chinese Medicine Integrated Database [46] (TCMID, http://www.megabionet.org/tcmid/), which records a large amount of information regarding formulas and their herbal ingredients; the TCM Database@Taiwan [47] (http://tcm.cmu.edu.tw/), which is the most comprehensive TCM database on the global scale; and the Traditional Chinese Medicine Systems Pharmacology Database [48] (TCMSP, http://lsp.nwu.edu.cn/), a unique system pharmacology platform devised for Chinese herbal medicines. Eventually, 69 herbal compounds were retrieved after deleting the duplicate data (Table S1).

### 2.2. Compound Targets for HDW

PubChem [49] (https://pubchem.ncbi.nlm.nih.gov/), as a public repository, provides information on chemical substances and their biological activities. We input all the active ingredients into PubChem and TCM Database@Taiwan and got the 3D molecular structure files of all active compounds in HDW. Because the targets of the compounds without precise structural information cannot be successfully predicted, we decided to remove these chemicals after deleting the replicate data. Eventually, 43 herbal compounds with structural information were reserved for further study. We imported these 3D molecular structure files into PharmMapper [50] (http://lilab.ecust.edu.cn/pharmmapper/), which is an online server that exploits pharmacophore mapping approach for potential drug target identification. The compounds without relevant 3D molecular structures information were removed. The top thirty targets of each compound acquired from PharmMapper were selected as potential targets in the present study. Thus, we collected distinct targets related to the compounds in HDW after discarding duplicate data (Table S2).

### 2.3. CRC Targets

The different genes associated with CRC were gathered from DisGeNET [51] (http://www.disgenet.org/), a comprehensive discovery platform developed for addressing diverse questions concerning the genetic underpinning of human diseases. We searched the platform with keywords “colorectal cancer” and selected 14 genes with the Gene-Disease Score >0.1. The details about the selected genes are described in Table S3.

### 2.4. Protein-Protein Interaction Data

The data of protein-protein interaction (PPI) came from String [52] (https://string-db.org/, ver. 10.5), with the species limited to “*Homo sapiens*.” String is a database of known and forecasted protein-protein interactions, and it defines PPI with confidence ranges for data scores (low confidence: scores <0.4; medium: 0.4 to 0.7; high: >0.7). Based on these scores, PPIs with comprehensive scores >0.7 were reserved in this study.

### 2.5. Network Construction

Network construction was performed as follows: (1) compound-compound target network was built by connecting chemical compounds and corresponding targets; (2) CRC targets' PPI network was established by linking 14 CRC targets retrieved from DisGeNET and other human proteins that directly or indirectly interacted with the 14 CRC targets; (3) compound-compound target-CRC target-other human proteins' PPI network was constructed by connecting compounds, intersection targets between compound targets and CRC targets' PPI network, and other human proteins that directly or indirectly interacted with the intersection targets.

The network visualization software Cytoscape [53] (http://cytoscape.org/, ver. 3.5.1) was adopted to present all of the above networks. The software is perfectly suitable for visualizing networks of intermolecular interactions, biological pathways, and many more. Besides, it provides a powerful set of data integration, analysis, and visualization functions to analyze complicated networks. For each node in the interaction network, three indices were calculated to evaluate its topological features. “Degree” is defined as the number of edges to node *i*; “Node betweenness” represents the number of shortest paths between pairs of nodes that run through node *i*; “Closeness” is the inverse of the sum of the distance from node *i* to other nodes.

### 2.6. Gene Ontology and Pathway Enrichment

The Database for Annotation, Visualization and Integrated Discovery [54] (DAVID, https://david.ncifcrf.gov/, ver. 6.8), which refers to a comprehensive set of functional annotation tools for understanding the biological meanings behind large gene datasets, was applied to perform Gene Ontology (GO) and Kyoto Encyclopedia of Genes and Genomes (KEGG) pathway enrichment analysis. Enriched GO terms and pathways were defined as those with False Discovery Rate (FDR) <0.01. In KEGG enrichment analysis, the bubble chart was plotted by using the OmicShare tools (http://www.omicshare.com/tools), a free online platform for data analysis.

## 3. Results and Discussion

### 3.1. Compound-Compound Target Network Analysis

**Figure 1:**
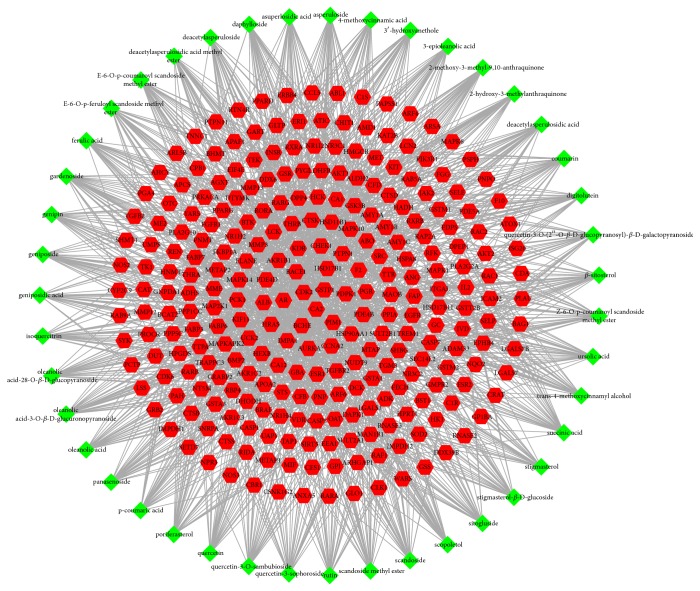
Compound-compound target network (green diamonds represent compounds contained in HDW. Red hexagons represent compound targets).

The compound-compound target network was depicted in Figure 1, including 309 nodes (43 active compound nodes and 266 compound target nodes) and 1260 edges. In this network, targets in the interior circle showed more interactions with compounds than those in the exterior. We found out that many targets were hit by multiple compounds. For instance, CA2 and GSTP1 were modulated by multiple ingredients including asperuloside, geniposide, and sitogluside. Also, CDK2, AR, and PDPK1 can also be regulated by more than one ingredient. This fact implied that the active chemicals of HDW might affect these targets synergistically and therefore have therapeutic effects on other diseases in addition to CRC, which virtually showed the properties of multicomponent, multitarget, and multidisease of the herbal medicine. Consequently, we could not only obtain an approximate observation of the relationship between bioactive compounds and compound targets but also discover the potential pharmacological effects of HDW from this network.

### 3.2. CRC Targets' PPI Network Analysis

**Figure 2:**
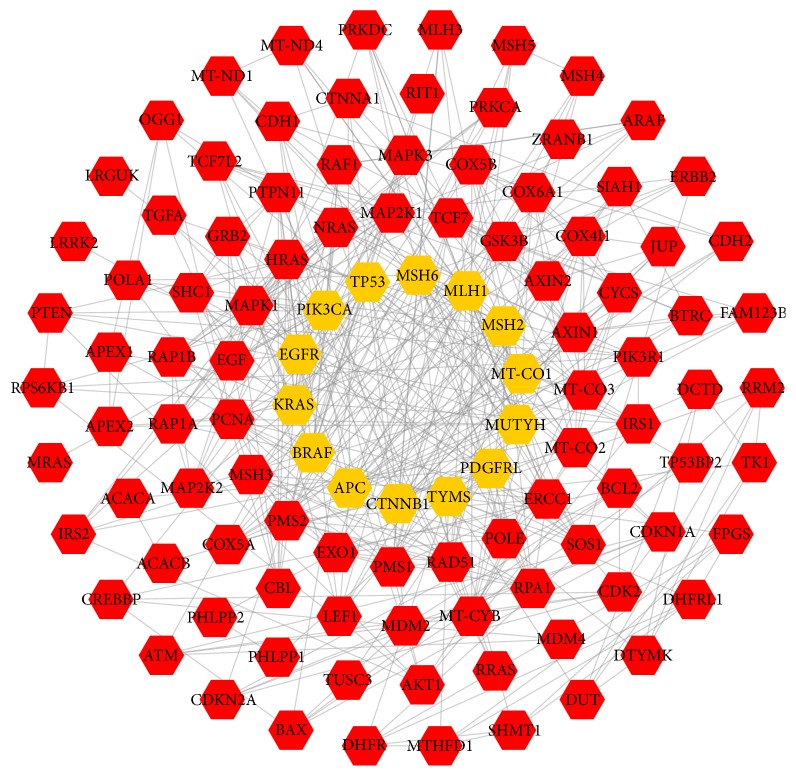
CRC targets' PPI network (orange hexagons represent targets related to colorectal cancer acquired from DisGeNET. Red hexagons represent other human proteins that are directly or indirectly interacting with the CRC targets).

The CRC targets' PPI network was shown in Figure 2, including 110 nodes (14 CRC target nodes and 96 other human protein nodes) and 428 edges. Three topological features of each node in the network were calculated to find the major nodes. Finally, 17 nodes with an average value of degree ≥9.63, node betweenness ≥0.037326, and closeness ≥0.6114 were selected as major nodes (Table S4), namely, PCNA, MSH2, MLH1, MSH6, PMS2, PMS1, PIK3CA, KRAS, HRAS, APC, CTNNB1, AXIN1, TYMS, MT-CO2, MT-CO3, MT-CO1, and MUTYH. Thus, these genes were likely to be the key or central genes in the development of CRC.

### 3.3. Compound-Compound Target-CRC Target-Other Human Proteins' PPI Network Analysis

**Figure 3:**
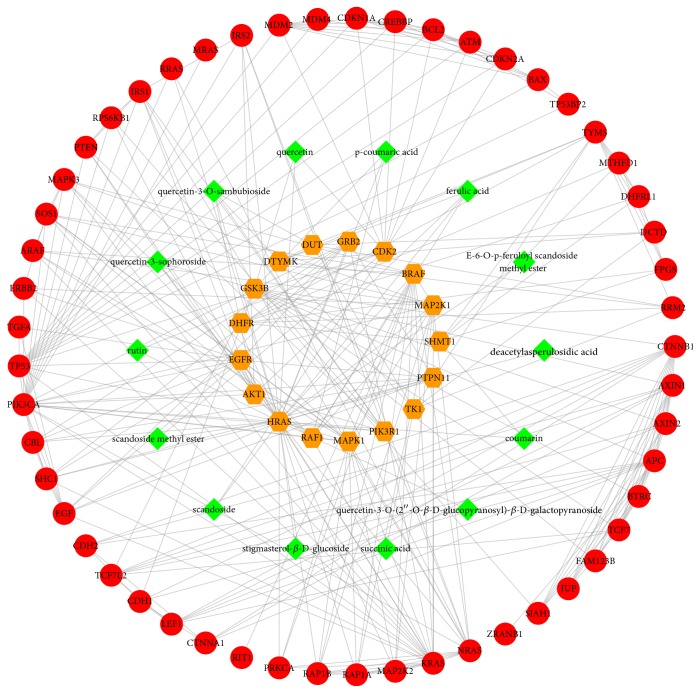
Compound-compound target-CRC target-other human proteins' PPI network (green diamonds represent compounds that have effects on intersection targets between compound targets and CRC targets. Orange hexagons represent intersection targets between compound targets and CRC targets. Red circles represent other human proteins that are directly or indirectly interacting with the intersection targets).

To analyze the significance of compound targets, a compound-compound target-CRC target-other human proteins' PPI network was constructed with 84 nodes (14 compounds, 17 intersection targets between compound targets and CRC targets' PPI network, and 53 other human proteins interacting with the intersection targets) and 306 edges (Figure 3). The topological features of the nodes were exhibited in Table S5; this provided us with a straightforward concept to distinguish those highly connected key nodes from the others in the network. The results of network analysis show that 10 nodes with an average value of degree ≥7.29, node betweenness ≥0.027360, and closeness ≥0.3208 could be considered as major nodes, including GTPase HRas (HRAS), phosphatidylinositol 4,5-bisphosphate 3-kinase catalytic subunit alpha isoform (PIK3CA), GTPase KRas (KRAS), cellular tumor antigen p53 (TP53), adenomatous polyposis coli protein (APC), serine/threonine-protein kinase B-raf (BRAF), glycogen synthase kinase-3 beta (GSK3B), cyclin-dependent kinase 2 (CDK2), RAC-alpha serine/threonine-protein kinase (AKT1), and RAF protooncogene serine/threonine-protein kinase (RAF1).

As we know, HDW probably exerts its therapeutic effect on CRC by binding and regulating particular protein targets. We speculated that the top 10 nodes might be the vital targets in the treatment of CRC. Consider GSK3B, PIK3CA, AKT1, RAF1, and CDK2. GSK3B was simultaneously targeted by 3 active chemicals: quercetin,* p*-coumaric acid, and quercetin-3-sophoroside. Glycogen synthase kinase-3 beta (GSK3B), a serine/threonine protein kinase encoded by* GSK3B*, has been acknowledged as a potential therapeutic target for multiple human cancers [55]. *β*-Catenin holds a vital status in Wnt/*β*-catenin pathway due to the fact that it can facilitate the transcription of several carcinogenic genes associated with cancer progression [56]. Previous relevant studies have defined that *β*-catenin may contribute to the development of cancer and is activated in 80% of colorectal cancers [57–60]. GSK3B shows the dual activity of inhibiting or promoting tumor [61, 62]. On one hand, since GSK3B serves as a negative regulator in the Wnt signaling pathway, it is considered to the tumor suppressor generally. The ubiquitin-mediated degradation of *β*-catenin occurs when GSK3B phosphorylates *β*-catenin in the Wnt signaling cascade. Consequently, the nuclear translocation and subsequent transcription of protooncogenes controlled by *β*-catenin are cleaved [63]. On the other hand, in light of recent evidence, GSK3B can activate the NF*κ*B signaling cascade through strengthening the transcriptional activity of NF*κ*B in the nucleus, which thereby promotes cancer [61]. Besides, it has been demonstrated that the suppression of the expression of GSK3B possibly abrogates tumor growth and induces apoptosis of CRC cells [62, 64]. Thus, the ingredients from HDW interacting with GSK3B may be the key factors in the treatment of the abnormal activation of *β*-catenin and NF*κ*B in patients with CRC. Meanwhile, other researchers have confirmed that HDW can increase the phosphorylation of *β*-catenin so as to inhibit the growth of CRC cells and CRC stem cells [65]. As for* PIK3CA*, its mutations are present in approximately 15 to 20% of colorectal cancers [66]. The phosphatidylinositol-3-kinase (PI3K) encoded by* PIK3CA *is a lipid kinase, and it plays a crucial role in promoting and regulating the signaling pathways associated with cell proliferation, migration, survival, apoptosis, and metabolism [67–69]. Mutations in the* PIK3CA* gene can initiate the constitutive activation of PI3K/AKT/mammalian target of rapamycin (mTOR) pathway, resulting in carcinogenesis and tumor progression [70–72]. Meanwhile, upregulation of PI3K enhances prostaglandin-endoperoxide synthase 2 activity and prostaglandin E_2_ synthesis, which inhibits the apoptosis of CRC cells [73]. In the PI3K/AKT/mTOR pathway, AKT, also known as protein kinase B (PKB), is a downstream effector of PI3K and is directly activated by it [69]. AKT1 as one of AKT family members was predicted to correlate with 3 active ingredients: quercetin-3-O-sambubioside, ferulic acid, and quercetin-3-sophoroside. The AKT family members are implicated in numerous cellular processes, including cell growth, proliferation, migration, metabolism, survival, and angiogenesis [67]. AKT overexpression has been proposed to be an early event in colorectal carcinogenesis [74]. AKT activates a series of downstream factors by phosphorylation and therefore modulates cellular metabolism that is rewired in cancer cells [75]. In general, our result suggested that key bioactive ingredients of HDW may produce therapeutic effects by inhibiting PIK3CA and AKT1 expression. Fortunately, in agreement with the findings of our research, previous findings have proven that 4-vinylphenol extracted from HDW can significantly downregulate PI3K and AKT expression in human endothelial cells [76]. In our study, we discovered that* p*-coumaric acid and ferulic acid can affect the activity of RAF1. RAF1 is a central member downstream of growth factors and RAS [77]. Overexpression of RAF1 facilitated the proliferation and invasion capacity of CRC cells [78]. Our work indicated that HDW might treat CRC by decreasing the expression of RAF1. With regard to CDK2, it was targeted by 4 active compounds from HDW: quercetin, rutin, scandoside methyl ester, and scandoside. CDK2 is an essential serine/threonine protein kinase mediating the cell cycle transition from G1 to S phase, and it thereby plays a key role in controlling cell proliferation [79–81]. CDK2 is often highly expressed in multiple malignant tumors, accelerating the cell cycle transition from G1 to S phase and thus promoting the proliferation of tumor cells [82, 83]. Accordingly, CDK2 expression levels have been reported to be higher in colorectal adenomas [84]. Our findings observed that HDW caused G1 cell cycle arrest by inhibiting CDK2 expression, producing the healing efficacy for CRC. Furthermore, other researchers have verified that HDW can significantly inhibit the proliferation of human hepatocellular carcinoma cells probably by restraining the activation of CDK2 [85].

### 3.4. GO and Pathway Enrichment Analyses

**Figure 4:**
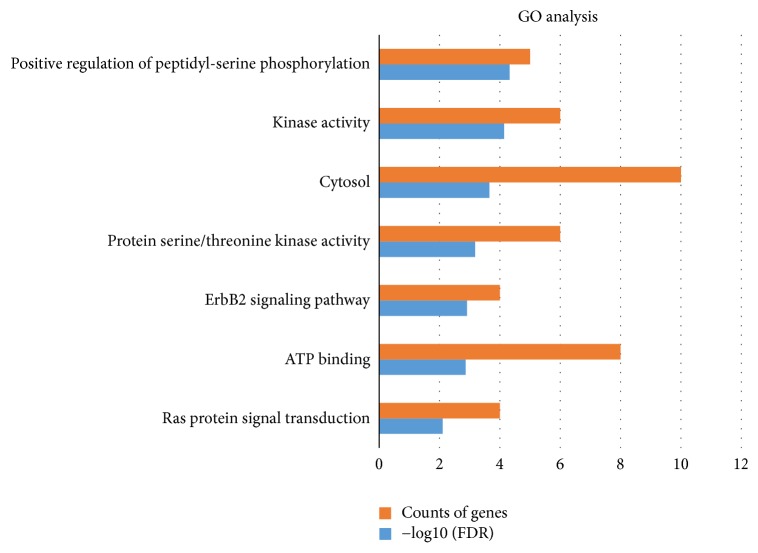
GO analysis for the major targets of HDW. The *y*-axis shows significantly enriched GO categories of the target genes, and the *x*-axis shows the enrichment scores of these terms or the counts of targets (FDR < 0.01).

To clarify the multiple mechanisms of HDW on CRC from a systematic level, we performed a GO enrichment analysis for the biological process, molecular function, and cellular component of the 10 selected targets.  Figure 4 listed the top 7 significantly enriched GO terms (FDR < 0.01) of these targets. *P* value and FDR were shown in Table S6. The results suggested that the targets of HDW were strongly correlated with 3 biological processes: positive regulation of peptidyl-serine phosphorylation, ErbB2 signaling pathway, and Ras protein signal transduction; 3 molecular functions: kinase activity, protein serine/threonine kinase activity, and ATP binding; and 1 cellular component: cytosol. This demonstrated that HDW probably worked by engaging in above biological processes, molecular functions, and cellular component.

**Figure 5:**
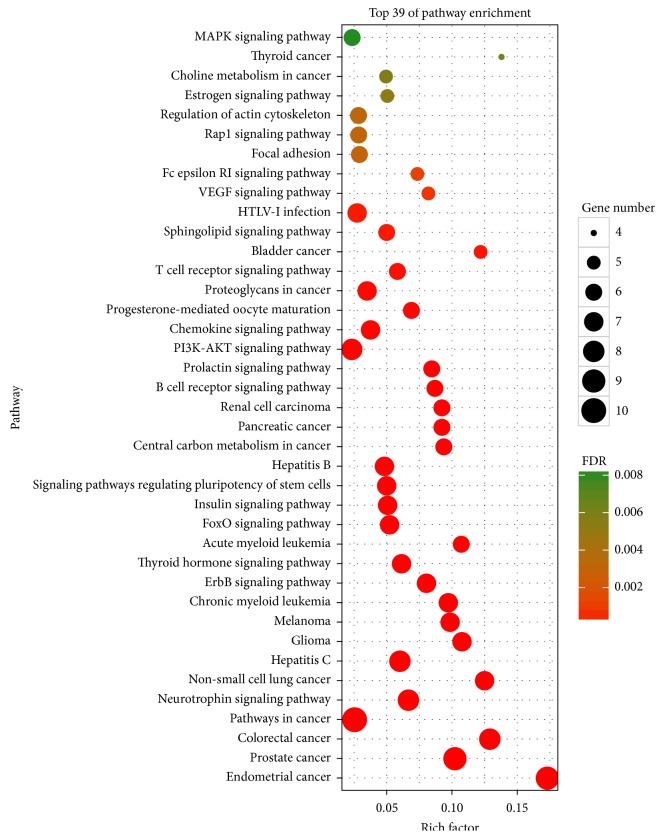
KEGG analysis for the major targets of HDW. The *y*-axis shows significantly enriched KEGG pathways of the target genes, and the *x*-axis shows the Rich factor (FDR < 0.01). Rich factor stands for the ratio of the number of target genes belonging to a pathway to the number of all the annotated genes located in the pathway. The higher Rich factor represents the higher level of enrichment. The size of the dot indicates the number of target genes in the pathway, and the color of the dot reflects the different FDR range.

As shown in Figure 5 and Table S7, the 10 targets were further mapped to 39 pathways with FDR <0.01. The 39 pathways belonged to four categories: human diseases (19/39), organismal systems (10/39), environmental information processing (6/39), and cellular processes (4/39). Thus, our findings showed that HDW integrated multiple signaling pathways to modulate cancers, infectious diseases, endocrine system, immune system, nervous system, signal transduction, cellular community, and cell motility. In addition, some pathways like colorectal cancer (hsa05210), pathways in cancer (hsa05200), PI3K-AKT signaling pathway (hsa04151), and MAPK signaling pathway (hsa04010) have been testified as accurate target pathways for curing CRC [86, 87]. We can also find that nearly half signaling pathways significantly enriched by targets were associated with multiple cancers, not merely CRC. The result indicated that HDW had the potential to treat diverse cancers, like prostate cancer, acute myeloid leukemia, pancreatic cancer, and bladder cancer, which has been confirmed by existing studies [88–91].

Among 39 signaling pathways, colorectal cancer (hsa05210) as the most important one regulates the process of apoptosis, proliferation, survival, and genetic stability for CRC cells. For instance, the decisive factors contributing to the initiation and evolution of CRC include the inactivation of tumor suppressor genes* APC* and* TP53* and the activation of the oncogene* KRAS* in colorectal cancer signaling pathway [92]. With respect to* APC*, 80% of colorectal cancers harbor inactivating mutations in* APC* gene [93], and APC inactivation is regarded as the initiating event in most colorectal cancers [94]. One of the crucial reasons responsible for the occurrence and development of CRC is the aberrant activation of Wnt/*β*-catenin signaling [95, 96]. Fortunately, the main biological function of APC in CRC is negatively regulating the Wnt signaling pathway by its interaction with *β*-catenin [97].* APC *mutations probably make the transcription of oncogenes such as c-myc and cyclin D1 unregulated, which in turn promotes tumorigenesis [98]. Notably, our research indicated that HDW was predicted to increase APC activity. Moreover, some evidence also shows that HDW can inhibit the growth of CRC cells and CRC stem cells by upregulating the expression of negative regulator APC [65]. When it comes to* TP53*, it is mutated in about 50% of patients with CRC [99], and mutations in* TP53* are considered to be relatively late events in the development of CRC [100]. The p53 protein encoded by* TP53* shows cancer-combating properties by initiating processes of cell cycle arrest, death, repair, or antiangiogenesis [101]. Importantly p53 DNA mutations destroy the tumor suppressor function of p53 and endow mutant p53 with a gain-of-function (GOF) to make it become a protooncogene [102, 103]. The GOF of mutant p53 subsequently brings about unfavorable events such as tumorigenesis, tumor progression, and drug resistance [103]. Although strenuous efforts have been contributed to regain the activity of p53 in treatments for cancer patients [104–108], the effectual clinical approaches developed based on p53 have failed to be discovered owing to the intricacy of p53 signaling [109]. Fortunately, our result suggested that HDW probably played a vital role in recovering p53 tumor suppressor activity. With regard to* KRAS*, its mutations are found in approximately 30–40% of colorectal cancers [110], and* KRAS* abnormalities can be detected early in the development of CRC [98]. Recent trends support that* KRAS* mutations have facilitated the cellular proliferation and malignant transformation of colorectal adenoma [111]. As for BRAF, it is a direct target of KRAS, and* BRAF* mutations are present in approximately 10% of CRC patients [112, 113].* KRAS* and* BRAF* are major oncogenic drivers of CRC [113]. BRAF and KRAS both activate the RAS/RAF/mitogen-activated protein kinase (MAPK) signaling pathway [112]. Previous studies have proven that the activation of MAPK signaling pathway shares the actions about directly affecting different cell cycle progression to involve the development and progression of CRC [114], and* KRAS* and* BRAF* mutations are the most frequently occurring alterations in the MAPK signaling cascade in CRC [113]. Moreover, the mutation status of* KRAS* and* BRAF* has been identified as predictive markers of resistance to epidermal growth factor receptor (EGFR) monoclonal antibody therapy in CRC [115, 116]. Based on the unfavorable fact, there is an urgent need for novel therapies to treat* KRAS* and* BRAF*-mutant colorectal cancers [112]. Interestingly, our findings implied that HDW produced the healing efficacy for CRC possibly by regulating KRAS and BRAF expression.

## 4. Conclusion

In our present study, we obtained 43 active ingredients from HDW and predicted 266 potential targets, suggesting that HDW was a complex agent that consisted of multiple components and affected numerous distinct targets. The network analysis uncovered that HDW probably exerted its pharmacological effects on CRC via modulating certain targets, including HRAS, PIK3CA, KRAS, TP53, APC, BRAF, GSK3B, CDK2, AKT1, and RAF1. The GO analysis of targets disclosed that the ingredients of HDW possibly produced synergistic effects for treating CRC mainly by regulating numerous biological processes, like regulation of peptidyl-serine phosphorylation, ErbB2 signaling pathway, and Ras protein signal transduction. Meanwhile, the pathway analysis in our work indicated that HDW might simultaneously act on diverse signaling pathways associated with the pathogenesis of CRC, including colorectal cancer (hsa05210), pathways in cancer (hsa05200), PI3K-AKT signaling pathway (hsa04151), and MAPK signaling pathway (hsa04010).

In summary, the current study is the first one that combines active ingredients, target prediction, network analysis, GO enrichment analysis, and pathway analysis by a network pharmacology method to illuminate the molecular and pharmacological mechanism of HDW against CRC from a systematic perspective. In this research, we showed here for the first time that HDW significantly affected multiple target genes mutated in patients with CRC, which was consistent with the recent trends that CRC can be attributed to the progressive accumulation of diverse genomic alterations in neoplastic cells [100]. Meanwhile, based on the systematic analysis for the bioactive compounds, crucial targets, and key pathways of HDW against CRC, our present study unveiled that the characteristics of HDW were multicomponent botanical therapeutics and multitarget synergetic therapeutic effects. Nonetheless, more experiments are warranted to verify the validity of our findings in further pharmacological and molecular research. Moreover, we hope that our study will be helpful for fostering novel research of other Chinese herbs against cancers and the application of network pharmacology for anticancer drug discovery context.

## Supplementary Materials

Supplementary Materials Table S1: the compounds information of HDW. Table S2: the compounds in HDW and their corresponding targets. Table S3: the targets of CRC. Table S4: the topological features of all nodes in the CRC targets' PPI network. Table S5: the topological features of all nodes in the compound-compound target-CRC target-other human proteins' PPI network. Table S6: the GO analysis for the major targets of HDW (FDR < 0.01). Table S7: the KEGG analysis for the major targets of HDW (FDR < 0.01).
